# COGNITIVE RESERVE ATTENUATES THE EFFECT OF AD GENETIC RISK ON COGNITIVE AND BRAIN TRAJECTORIES

**DOI:** 10.1093/geroni/igae098.2305

**Published:** 2024-12-31

**Authors:** Corinne Pettigrew, Anja Soldan, Jiangxia Wang, Alden Gross, Timothy Hohman, Christos Davatzikos, Guray Erus, Marilyn Albert

**Affiliations:** Johns Hopkins University School of Medicine, Baltimore, Maryland, United States; Johns Hopkins University, Baltimore, Maryland, United States; Johns Hopkins University, Baltimore, Maryland, United States; Johns Hopkins Bloomberg School of Public Health, Baltimore, Maryland, United States; Vanderbilt University Medical Center, Nashville, Tennessee, United States; University of Pennsylvania, Philadelphia, Pennsylvania, United States; University of Pennsylvania, Philadelphia, Pennsylvania, United States; Johns Hopkins University School of Medicine, Baltimore, Maryland, United States

## Abstract

Alzheimer’s disease (AD) genetic risk factors impact cognitive and brain health, but the extent to which these effects are mitigated by cognitive reserve (CR) is less well understood. This study examined whether higher levels of CR (measured by education and reading ability) were associated with a reduced impact of AD genetic risk on both longitudinal cognitive trajectories (N = 1,819, M follow-up = 9.9 years) and brain atrophy (N = 1,542, M follow-up = 5.3 years) measured in middle-aged and older individuals with normal cognition at baseline. AD genetic risk was measured by APOE genetic status and AD polygenic risk scores (AD-PRS, excluding APOE). Cognitive outcomes included global, episodic memory, and executive function factor scores. Atrophy, derived from volumetric magnetic resonance imaging, included a composite of AD vulnerable regions (SPARE-AD), hippocampal volume, and a composite of regions indexing advanced non-AD related brain aging (SPARE-BA). In mixed-effects models, APOE-ε4 was associated with declines in all cognitive measures and with greater atrophy in SPARE-AD, SPARE-BA, and hippocampal regions over time. Importantly, the negative associations of APOE-ε4 with declines in global cognition and memory, and of APOE-ε4 with atrophy in SPARE-AD and the hippocampus were reduced among individuals with higher levels of CR. The AD-PRS was associated with executive function and global cognition declines (not memory), and with greater atrophy in SPARE-AD and hippocampal regions. However, CR did not attenuate AD-PRS-related changes in these measures. Results suggest that some APOE-ε4-related declines in cognition and brain atrophy may be mitigated by potentially modifiable factors.

